# Comparison of rebubbling rate between preloaded endothelium-in and preloaded no-touch endothelium-out Descemet membrane endothelial keratoplasty transplantation

**DOI:** 10.1186/s12886-024-03560-0

**Published:** 2024-07-22

**Authors:** Martin Kronschläger, Alessandro Ruzza, Johannes Zeilinger, Andreas Schlatter, Manuel Ruiss, Oliver Findl

**Affiliations:** 1 Department of Ophthalmology, Vienna Institute for Research in Ocular Surgery (VIROS), Karl Landsteiner Institute, Hanusch Hospital, Heinrich Collin Strasse 30, Vienna, 1140 Austria; 2 Fondazione Banca degli Occhi del Veneto, ETS, Via Paccagnella n. 11 - Padiglione Rama, Zelarino, Venice, 30174 Italy

**Keywords:** Descemet membrane endothelial keratoplasty, DMEK, Preloaded, Endothelium-out, Endothelium-in

## Abstract

**Background:**

To compare the difference in rebubbling rates between patients undergoing Descemet membrane endothelial keratoplasty (DMEK) with endothelium-in using a standard IOL cartridge and those with endothelium-out DMEK utilizing a no-touch technique with borosilicate glass cartridge transplantation.

**Methods:**

This retrospective study included all eyes that underwent preloaded endothelium-in or endothelium-out DMEK transplantation from June 2019 to December 2023 at the Hanusch Hospital, Vienna, Austria. All DMEKs were harvested, prepared and preloaded at the European Eye Bank of Venice, Italy. DMEK surgeries were done by one experienced surgeon and the procedure was completed by air tamponade of the anterior chamber.

**Results:**

Overall, 32 eyes each of 31 endothelium-out patients and of 29 endothelium-in patients were included. 32 preloaded endothelium-in procedures were followed by 32 preloaded endothelium-out procedures. Rebubbling rate for endothelium-in was 15/32 (47%) and for endothelium-out was 7/25 (28%) (*p* = 0.035, Pearson’s chi-squared test). Donor age was the most important variable for rebubbling in a random forest algorithm model (ROC: 0.69).

**Conclusions:**

Rebubbling rate in endothelium-out DMEK was less than two-thirds compared to endothelium-in DMEK favoring no-touch endothelium-out DMEK as the preferred technique of DMEK transplantation.

## Background

The endothelium, which is the innermost layer of the cornea, can be compromised either by different diseases, of which Fuchs endothelial corneal dystrophy (FECS) is the most prevalent, or through insults like surgeries, infections, traumas, etc. All of these events can disturb the hydration of the tissue resulting in reduction of transparency and visual impairment [1].

To treat this condition, posterior lamellar keratoplasty (PLK) can be used, of which the two main procedures are Descemet stripping automated endothelial keratoplasty (DSAEK) and Descemet membrane endothelial keratoplasty (DMEK) [2, 3]. While for the first intervention a microkeratome is used to gain donor tissue containing endothelium, Descemet membrane, and a posterior part of the stroma of the cornea, in the latter procedure a thinner graft consisting of only the endothelium and the Descemet membrane is manually prepared [4, 5]. According to the literature, PLK has some advantages compared to penetrating keratoplasty (PKP), which is full thickness corneal transplantation, for example more rapid and better visual acuity outcomes, smaller amount of postoperative astigmatism, decreased rate of endothelial cell (EC) loss, and a reduced graft rejection rate [2, 6–8].

When comparing the techniques mentioned above, DSAEK and DMEK, it was shown that the latter one had lower rates of posterior corneal higher order aberrations (HOAs), better visual outcomes, lower graft rejection rates, less cell density loss, and higher patient’s satisfaction [2, 9, 10]. On the other hand, DMEK seems to be technically more difficult and, hence, harder to learn and a 2.5x higher rebubbling rate due to graft detachment was reported [2, 10–12]. Rebubbling is associated with significantly higher endothelial cell loss, i.e. a higher risk for graft failure [13].

One way to reduce detachment of the graft and, hence, the rebubbling rate, would be to improve the preparation as well as the handling of the donor tissues. This would prevent loss of corneal ECs induced by mechanical damage leading to long-term graft survival [14]. The isolated Descemet membrane has the tendency to roll in a fashion that the endothelium is on the outside (endothelium-out). This configuration may expose the ECs to potential damage during the loading or insertion process through contact with the injector or the cartridge [15, 16]. One way to avoid this, is to scroll the tissue in such a way that its endothelium is exposed to the inside (endothelium-in), whereby potential damage to ECs is reduced [16]. Another option to prevent EC damage would be that only trained personnel, e.g. from an eye bank, prepares and preloads the donor tissue under standardized and quality controlled conditions [17].

The aim of this study is to compare the rebubbling rate in patients that had DMEK with a non-touch preloaded endothelium-out borosilicate glass cartridge with those in which a standard cartridge endothelium-in was used during the intervention.

## Methods

### Study participants

This single-center retrospective study included patients who underwent endothelium-in or endothelium-out DMEK from June 2019 to December 2023 at the Department of Ophthalmology of the Hanusch Hospital in Vienna, Austria. Excluded were cases with any kind of gas fill and cases with intraoperative complications. All procedures involving patients were performed in accordance with the Declaration of Helsinki and were approved by the local ethical committee of the city of Vienna (EK 22-130-VK). All corneas used were recruited and processed by the Fondazione Banca degli Occhi del Veneto (FBOV) according to the guidelines of the National Transplant Center (NTC), Italy.

### Data collection

The medical files of all eligible patients were reviewed and pseudonymized. The following demographic and preoperative information was collected: age, gender, pathology of patient cornea, orientation of the transplant in the preloaded device, transplant endothelial cell count, age of donor, sex of donor, date of donor death, date of transplant excision, date of corneoscleral rim isolation. The following postoperative information was collected: date of transplantation, rebubbling and combined cataract and DMEK surgery, All data were handled completely pseudonymized.

### Stripping procedure

All the tissues used in this study were preserved in Organ culture i.e. in tissue culture medium (TCM) at 31 °C. To prepare the DMEK grafts, the corneas were de-swollen for 24–48 h in the same TCM supplemented with 6% (wt/vol) dextran T-500. The corneas were centered with the endothelium facing up on a vacuum base and the suction was created by a syringe. A 9.5-mm blade punch (Moria, Antony, France) was used to make a superficial incision through the Descemet’s membrane. The circular wound was stained with trypan blue for 20 s to better visualize the excised area and the peripheral membrane was removed. The endothelium was kept moist throughout the procedure using PBS or transport medium (TM - tissue culture media supplemented with 6% dextran). The peripheral membrane was carefully lifted up by a Fogla hook (Janah s.r.l., Como, Italy) to find out the right cleavage plain, i.e. between Descemet’s membrane and stroma. The periphery of the membrane was grabbed using a tying forceps (Janah s.r.l., Como, Italy) and stripped leaving an 1,5 –2 mm of adhesion area between Descemet and stroma. On the naked portion of the stroma, a 2 mm dermal punch was used to obtain a full thickness stromal biopsy. The membrane was placed back on the stromal bed, the liquid was carefully removed and the cornea placed upside down with the epithelium facing up. The stromal biopsy was removed and an “F” letter was drawn using the tip of the Fogla hook, stained with a skin marker.

The membrane was stained with 0,2% trypan blue for 45 s and gently washed with PBS. The endothelial cells were exposed to 1.8% sucrose solution inside a Petri plate to assess cell density and mortality under inverted light microscope (Axiovert, Zeiss, Milan, Italy). The cells were manually counted using a 10 × 10 mm in-built reticule inside the eyepiece. An average of 5 counts was performed throughout the endothelial surface to obtain the density per square millimeter.

### Loading procedure

The corneas were replaced on a vacuum block to resize the diameter of the membranes to 8 mm using a trephine. The grafts were processed following two different procedures based on the type of devices where they were loaded [16, 18].

**Fig. 1:**
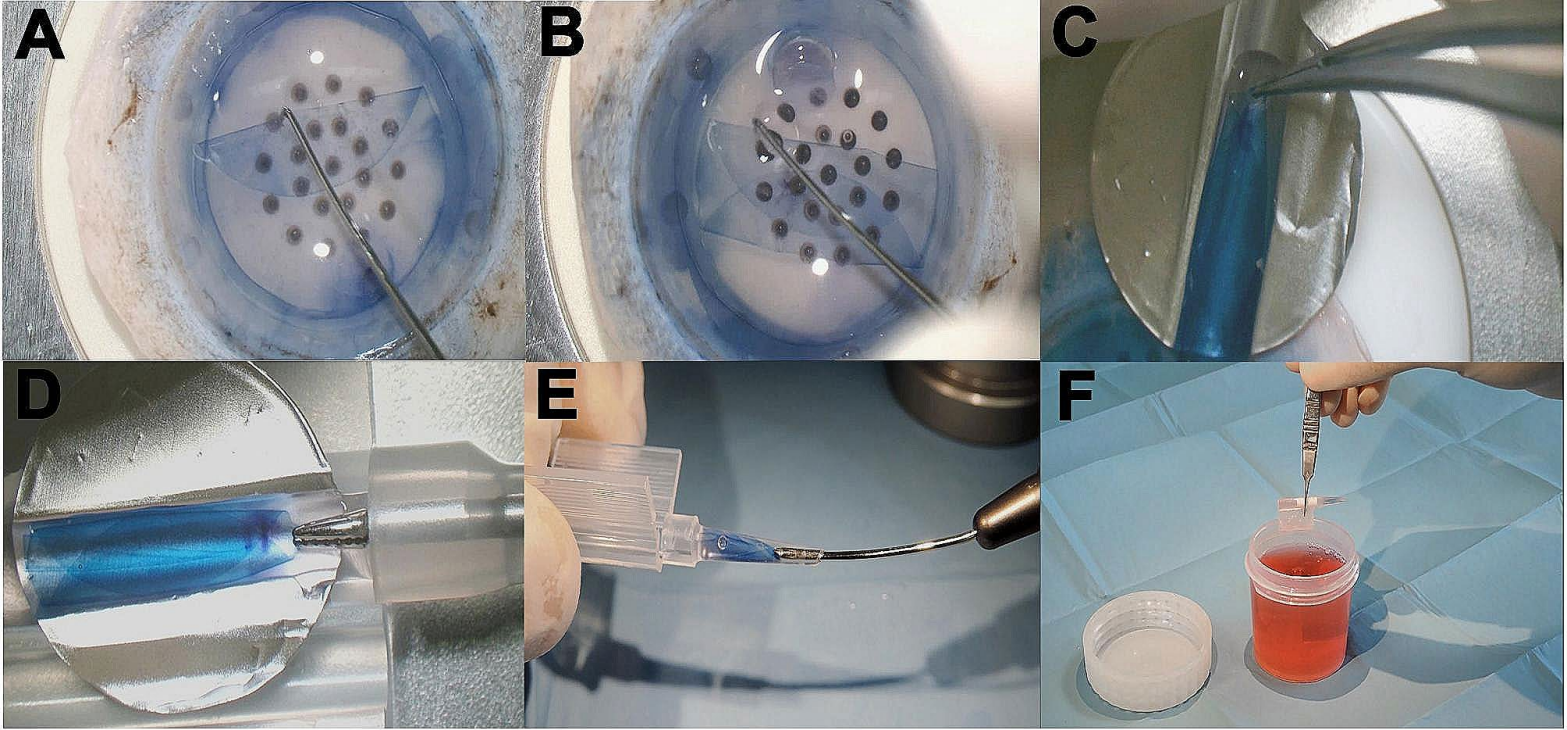
Endothelium-in system: **A** + **B**: tri-folding. **C**: mounting on aluminum disc. **D** + **E**: loading in the cartridge. **F**: inserting the cartridge in the container

ENDOTHELIUM-IN system (Fig. 1).

A small drop of TM was added on the endothelium to keep it moist during the entire procedure. The periphery of the membrane was grabbed from two ends using an acute forceps (Janach s.r.l., Como, Italy) and tri-folded, with the endothelium inward configuration. The membrane was then stained with a few drops of trypan blue for 20 s to enhance its visualization during loading phase. A sterile 11 mm aluminum disk was shaped on the base of the groove of the IOL cartridge (MDJ sas, La Monnerie-LeMontel, France) and placed on the corneo-scleral rim, close to the folded membrane. The concavity of the molded disk was filled with TM and the graft was slid over it, taking care to maintain its tri-folded configuration. The aluminum disc was transferred on the iol cartridge which was filled with TM and the membrane loaded inside the funnel through the exit hole using a 23G TWEEZER grip forceps (Aktive Srl, Roma, Italy). A stopper was applied to the back part of the cartridge to prevent displacement of membrane in the posterior segment of the device. Once closed, the cartridge was preserved in a sterile bottle filled with TM and stored in an incubator at 31 °C. A microbiology test was performed before the shipment.

**Fig. 2:**
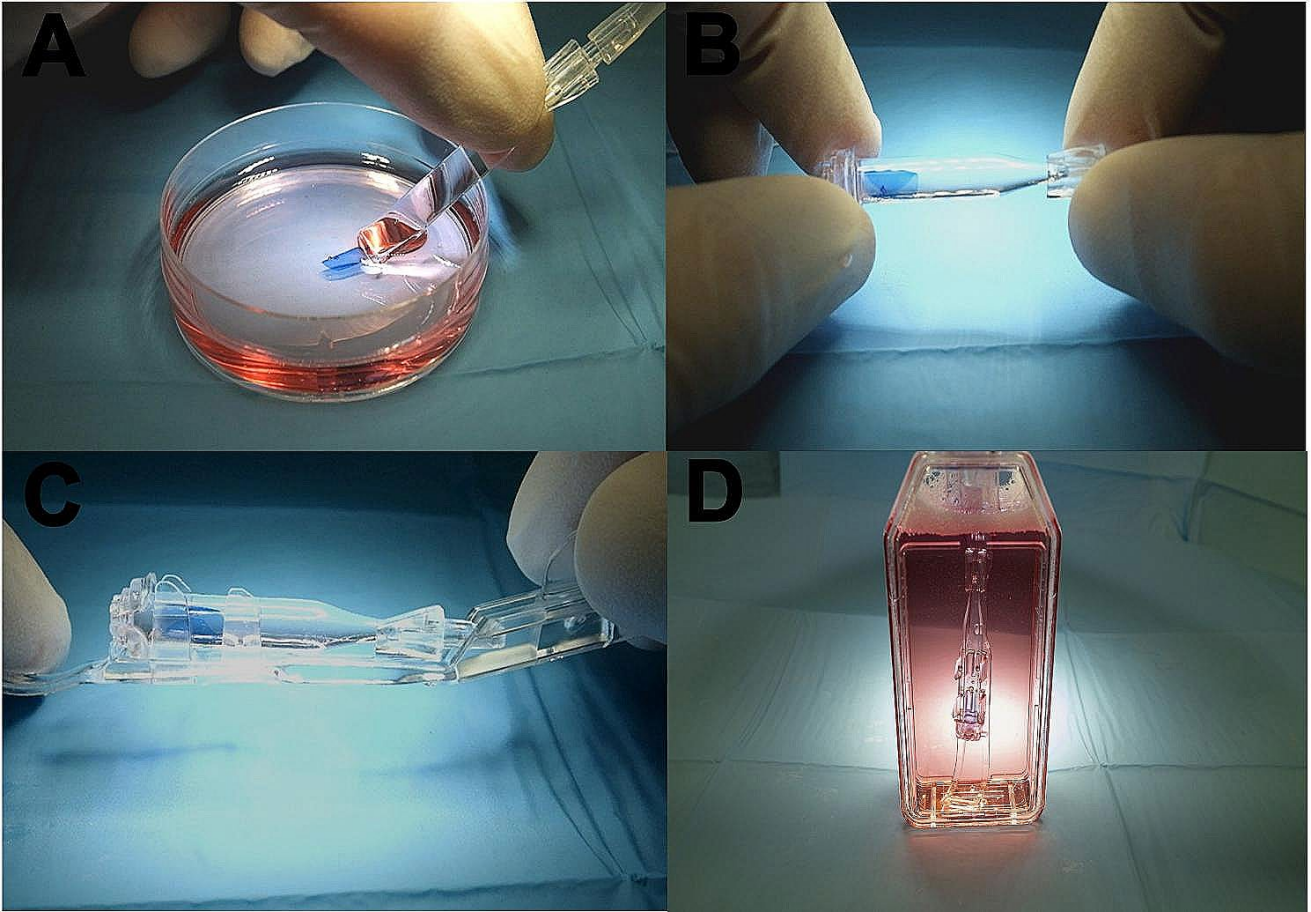
Endothelium-out system: **A**: no-touch loading. **B**: closing the cartridge. **C**: fixing the cartridge in the holder. **D**: cartridge in the flask

ENDOTHELIUM-OUT system (Fig. 2).

The membrane was gently detached from its base and re-stained with trypan blue for 20 s. The graft was transferred in a petri plate filled with TM, allowing to obtain its natural configuration with the endothelium outwards. The DMEK Rapid device (GEUDER, Heidelberg, Germany) was connected to the anterior plug where the silicone tube was attached together with a 5 mL syringe. The whole system was filled with TM, preventing the formation of air bubbles, and the graft was aspirated from the petri plates into the cartridge. The back stopper was fixed on the cartridge and the syringe with the silicone tubing was detached from the anterior plug. The entire device was secured into the holder and placed in a flask (Gibco, NY), filled with TM medium. The system was preserved in an incubator at 31 °C and shipped after microbiology control.

### Surgical technique

All surgeries were performed by one experienced DMEK surgeon (O.F.) in a similar fashion under neuroleptic anesthesia with sub-tenon anesthesia. All patients received a YAG iridotomy at 6 o’clock at least 2 weeks before surgery. Ink-marked calipers were used to mark the corneal diameter of the descemetorhexis 8.5 mm. The preloaded DMEKs were re-stained with trypan blue. A 2.8 mm limbal incision and 2 paracentesis were created and an anterior chamber maintainer was placed. Descemetorhexis was performed using a reverse Sinskey hook. In case of late graft failure the endothelial transplant was removed with a reverse Sinskey hook. The preloaded grafts were inserted into the eye and the anterior chamber maintainer was removed. After unfolding and centration of the graft the eye was completely filled with air. The eye was kept with a 100% air fill and slightly above physiological pressure (according to surgeon touch).

### Combined surgical technique

Patients with cataracts were operated with a combined surgical technique. A standard cataract surgery was performed using Healon OVD (Johnson&Johnson, USA) for capsulorhexis and IOL implantation. After implanting the IOL in the bag a 8.5 mm descemetorhexis was performed with Healon OVD in the anterior chamber. Then the Healon OVD was removed by meticulous irrigation/aspiration and the DMEK surgical technique was continued as described above.

### Rebubbling

The rebubbling indication was set on a proactive stance. Rebubbling was performed even in cases of slight peripheral detachment.

### Statistical analysis

Data were analyzed with Microsoft Excel 2021, R 4.3.2. and SPSS Statistics 29. All presented means are accompanied by their respective standard deviations. The significance limit was set to 0.05 and 0.95, respectively.

## Results

From June 2019 to July 2021, we performed 32 preloaded endothelium-in DMEK transplantation of 29 patients followed by 32 preloaded endothelium-out DMEK transplantation of 31 patients with the DMEK rapid device from September 2021 to December 2023 all prepared by the same corneal bank. No cases were excluded because of primary gas fill or complications within surgery. Indications for DMEK were Fuchs endothelial dystrophy (endothelium-in: 29/32, 91%; endothelium-out: 22/32, 69%), bullous keratopathy (endothelium-in: 1/32; 3%; endothelium-out: 4/32, 12%) and late graft failure (endothelium-in: 2/32, 6%; endothelium-out: 6/32, 19%). Detailed patient characteristics are shown in Table 1. Endothelial cell count before surgery of the tx was 2560 ± 140 (cells/mm^2^) for endothelium-out and 2560 ± 180 (cells/mm^2^) for endothelium-in. (Table 2) 11 DMEK transplantations of each group were combined surgeries, i.e. including cataract surgery. (Table 3). Gender match (donor- recipient) of the endothelium-in group was 15/32 (47%) and of the endothelium-out group was 13/32 (41%) and days from donor death to transplantation was 17 ± 4 for the endothelium-in group and 14 ± 2 in the endothelium-out group (Table 3).

**Table 1:** Patient characteristics of the endothelium-in and endothelium-out group. Age was described as mean and standard deviation

	female (*n*)	male (*n*)	age (years)	endothelial dystrophy	bullous keratopathy	late graft failure
endo-in	14	18	70 ± 11	91%	3%	6%
endo-out	20	12	72 ± 10	69%	12%	19%

**Table 2:** Transplant characteristics of the endothelium-in and endothelium-out group. Age and endothelial cell count were described as mean and standard deviation

	female (*n*)	male (*n*)	age (years)	endothelial cell count (cells/mm^2^)	Donor death to excision of tissue (median days)
endo-in	13	19	67 ± 8	2560 ± 140	0
endo-out	9	23	69 ± 6	2560 ± 180	0

**Table 3:** Surgery characteristics of the endothelium-in and endothelium-out group. Donor death to transplantation, corneal rim isolation to transplantation and storage time of preloaded DMEK to surgery were described as mean and standard deviation

	combined cataract surgery(*n*)	gender match donor - host (*n*)	donor death to transplantation (days)	corneal rim isolation to transplantation (days)	storage time of preloaded DMEK within cannula (days)
endo-in	11	15	17 ± 4	17 ± 4	2 ± 1
endo-out	11	13	14 ± 2	14 ± 2	2 ± 1

### Rebubbling

In the endothelium-in group two patients had to be rebubbled two times, which we considered in this study as one rebubbling for each of the two patients. Rebubbling rate for endothelium-in was 15/32 (47%) and for endothelium-out was 7/25 (28%) (*p* = 0.035, Pearson’s chi-squared test). (Table 4) A random forest algorithm addressing rebubbling rate resulted in a receiver operating characteristic (ROC) = 0.69, sensitivity = 0.83 and specificity = 0.31. In this model the importance of variables is shown in Table 5. The importance of variables in decreasing order is: donor age, recipient age, time rim isolation to transplantation, gender mismatch, time donor death to transplantation, orientation of transplant, transplant endothelial cell count before surgery, pathology, time donor death to excision and sex of donor.

**Table 4:** Rebubble frequency between the endothelium-in and endothelium-out group

	rebubble (*n*)	no rebubble (*n*)
endo-in	15	17
endo-out	7	25

Pearson’s chi-squared test *p* = 0.035

**Table 5:** Generically calculated variable importance for the rebubble random forest algorithm (scaled 0–100)

variable	importance
donor age	100.00
recipient age	79.60
time rim isolation to transplantation	39.01
time donor death to transplantation	30.36
gender mismatch	25.54
tx endothelial cell count before surgery	20.22
pathology	18.67
orientation of tx	17.67
sex of recipient	10.53
time donor death to excision	7.39
storage time preloaded DMEK	3.37
sex of donor	0.5
combined cataract surgery	0

## Discussion

In this retrospective study, we demonstrate that endothelium-in DMEK transplantation resulted in significantly more rebubbling than no-touch endothelium-out DMEK transplantation. As previously described, time to unfold in endothelium-in DMEK is significantly shorter than in endothelium-out DMEK, consequently reducing overall surgical time and surgical manipulation. However, loading time for endothelium-in technique is significantly longer than in no-touch endothelium-out technique, though there is no significant difference in cell loss between both techniques [16]. We speculate that longer loading time might lead to tissue alterations which result in a higher rebubbling rate.

However, Price et al. found no difference in rebubbling rate comparing freshly prepared endothelium-in and endothelium-out DMEK. This was observed using the same IOL injector for loading, without employing a no-touch technique for endothelium-out DMEK loading [19]. In this surgeon stripped DMEK grafts Price et al. reports of rebubbling rates of only 10 to 13% in both groups. We found a much higher rebubbling rate of 47% in endothelium-in DMEKs and 28% in endothelium-out DMEKs. This could be attributed to our proactive stance on performing rebubbling, even for minor peripheral detachment instances, contrasted with Price et al.’s approach of reserving rebubbling for detachments impacting vision or exhibiting progressive enlargement. Nonetheless, the rebubbling rates of preloaded eye bank-prepared DMEKs in our study mirrored those reported by Romano et al. Their study documented rebubbling rates of 48%, 40%, and 15% for preloaded, prestripped, and surgeon-stripped DMEK grafts, respectively. Increased rebubbling rates in eye bank preloaded tissues might be due to decreased adhesion forces and elastic modulus [20], which were probably linked to storage time of preloaded DMEK tissue. All DMEKs were performed with air tamponade of the anterior chamber. A gas tamponade would have probably reduced rebubble rates [21]. Moreover, Romano et al. found that rebubbling rates were significantly corresponding with combined cataract surgery and time from harvesting the graft to the surgery [20]. In our study, we observed a similar amount of combined cataract and DMEK surgeries in both groups, i.e. 11 each. (Table 3) Time from harvesting of the graft to surgery and storage time of the preloaded DMEK within the cannula to surgery were without statistical significance between both groups. (Table 3) Recently, Tapley et al. found that rebubbling rates for Fuchs dystrophy were lower in Fuchs dystrophy (13%) than in failed penetrating keratoplasty (29%) and pseudophakic bullous keratopathy (28%) [22]. In our study, Fuchs dystrophy emerged as the predominant pathology in both endothelium-in and endothelium-out DMEKs. (Table 1) Despite encountering a higher incidence of rebubbling in endothelium-in DMEKs, our study revealed that this group comprised the highest number of Fuchs dystrophy patients compared to the endothelium-out DMEK group. (Table 4).

**Fig. 3:**
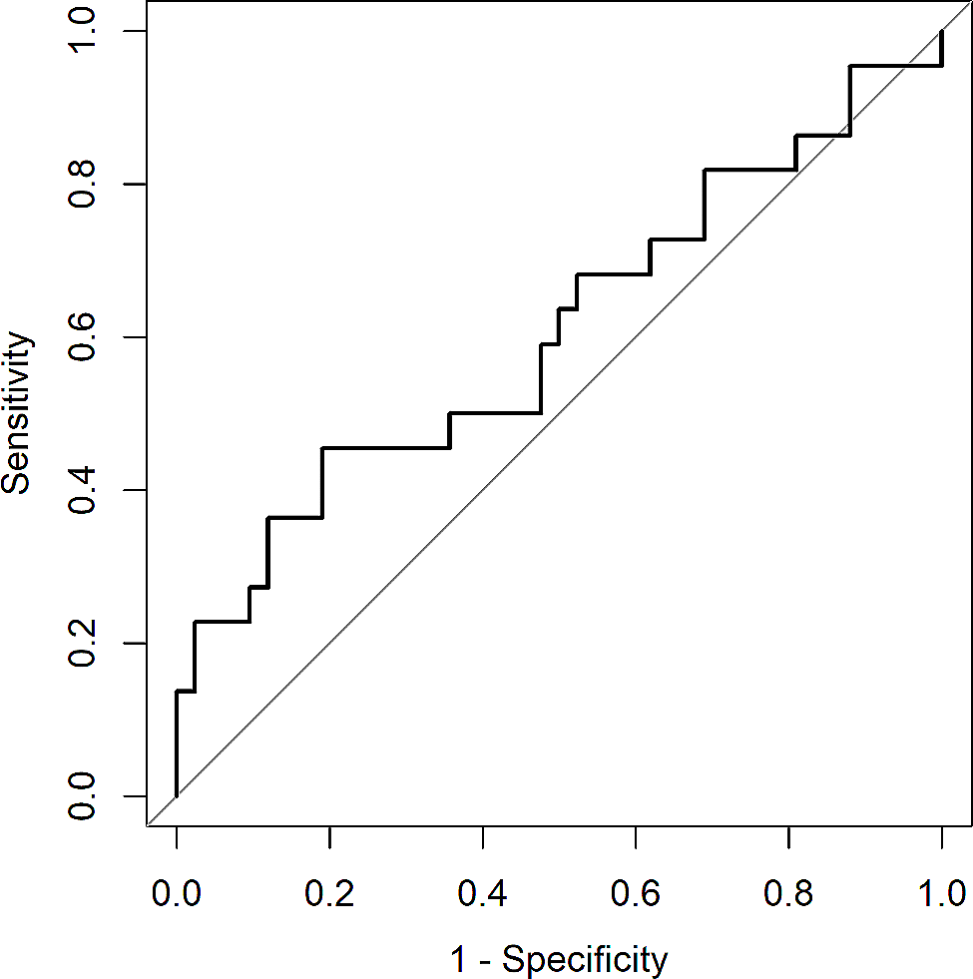
Receiver operating characteristic (ROC) curve on rebubbling across different thresholds. The boundary of no discrimination is shown in a grey line. The model characteristics: 5-fold cross-validated, ROC = 0.69, sensitivity = 0.83, specificity = 0.31, number of trees = 500

A random forest model of our data (Fig. 3) revealed donor age (Table 5) to be the most important variable for rebubbling. The random forest variable importance scores are aggregate measures. They only measure the influence of the predictor, not its precise effect. However, comparing donor age of the rebubbling and non-rebubbling group result in a Jaccard index of 0.48. Average donor age of the rebubbling group is slightly younger than in the non- rebubbling group indicating a younger donor age as risk factor for rebubbling. This is in contrast to recent studies which showed that donor age did not influence rebubbling rate [23, 24]. Consequently, the outcome might be a result of our mixed preloaded DMEK techniques.

Strengths of this study were the standardized preloading technique by one corneal eye bank, an uniform surgical technique by one surgeon and roughly similar cohorts. Study limitations included the retrospective nature, the lack of randomization, limited number of patients and missing postoperative endothelial cell count.

Rebubbling is a critical factor in DMEK outcome. The act of rebubbling led to diminished visual acuity and increased loss of endothelial cells [3, 13], underscoring the importance of considering these factors before choosing DMEK insertion technique.

## Conclusions

The rebubbling rate in preloaded no-touch endothelium-out DMEK was less than two-thirds compared to preloaded endothelium-in DMEK, indicating a preference for the preloaded no-touch endothelium-out DMEK technique in DMEK transplantation.

## Data Availability

The datasets used and/or analysed during the current study are available from the corresponding author on reasonable request.

## Abbreviations

DMEK: Descemet membrane endothelial keratoplasty
DSAEK: Descemet stripping automated endothelial keratoplasty
EC: Endothelial cell
FBOV: Fondazione Banca degli Occhi del Veneto
FECD: Fuchs endothelial corneal dystrophy
HOA: Higher order aberrations
NTC: National Transplant Center
PLK: Posterior lamellar keratoplasty
TCM: Tissue culture medium
TX: Transplant
